# Mechanism-guided engineering of a minimal biological particle for genome editing

**DOI:** 10.1073/pnas.2413519121

**Published:** 2024-12-30

**Authors:** Wayne Ngo, Julia Peukes, Alisha Baldwin, Zhiwei Wayne Xue, Sidney Hwang, Robert R. Stickels, Zhi Lin, Ansuman T. Satpathy, James A. Wells, Randy Schekman, Eva Nogales, Jennifer A. Doudna

**Affiliations:** ^a^Innovative Genomics Institute, University of California, Berkeley, CA 94720; ^b^Institute of Data Science and Biotechnology, Gladstone Institutes, San Francisco, CA 94158; ^c^California Institute for Quantitative Biosciences, University of California, Berkeley, CA 94158; ^d^Department of Molecular and Cell Biology, University of California, Berkeley, CA 94720; ^e^Department of Pathology, Stanford University, Stanford, CA 94304; ^f^Gladstone-University of California, San Francisco Institute of Genomic Immunology, San Francisco, CA 94158; ^g^Parker Institute for Cancer Immunotherapy, San Francisco, CA 94129; ^h^Department of Pharmaceutical Chemistry, University of California, San Francisco, CA 94158; ^i^Department of Cellular and Molecular Pharmacology, University of California, San Francisco, CA 94158; ^j^HHMI, University of California, Berkeley, CA 94720; ^k^Molecular Biophysics and Integrated Bioimaging Division, Lawrence Berkeley National Laboratory, Berkeley, CA 94720; ^l^Department of Chemistry, University of California, Berkeley, CA 94720

**Keywords:** genome editing, delivery, viral-like particles

## Abstract

The widespread application of genome editing to treat and cure disease requires the delivery of genome editors into the nucleus of target cells. Enveloped delivery vehicles (EDVs) are engineered virally derived particles capable of packaging and delivering CRISPR-Cas9 ribonucleoproteins (RNPs). However, the presence of lentiviral genome encapsulation and replication proteins in EDVs has obscured the underlying delivery mechanism and precluded particle optimization. Here, we show that Cas9 RNP nuclear delivery is independent of the native lentiviral capsid structure. Instead, EDV-mediated genome editing activity corresponds directly to the number of nuclear localization sequences on the Cas9 enzyme. EDV structural analysis using cryo-electron tomography and small molecule inhibitors guided the removal of ~80% of viral residues, creating a minimal EDV (miniEDV) that retains full RNP delivery capability. MiniEDVs are 25% smaller yet package equivalent amounts of Cas9 RNPs relative to the original EDVs and demonstrated increased editing in cell lines and therapeutically relevant primary human T cells. These results show that virally derived particles can be streamlined to create efficacious genome editing delivery vehicles with simpler production and manufacturing.

## Significance

Delivery of genome editing enzymes to diseased cells is critical for realizing their therapeutic potential. Virally derived particles, such as enveloped delivery vehicles (EDVs), use viral proteins for packaging and delivering editing enzymes. Understanding how they function is essential for improving editing enzyme delivery. We demonstrate that the nuclear delivery of Cas9 ribonucleoproteins via EDVs relies on engineered nuclear localization signals rather than the native viral capsid structure. By removing unnecessary viral components and improving nuclear localization, we engineered minimal EDVs (miniEDVs) that showed increased editing efficiency. Our findings highlight the importance of understanding how virally derived particles function to eliminate unnecessary viral proteins and create more efficacious and easier-to-produce delivery vehicles for therapeutic genome editing.

CRISPR-Cas9-mediated genome editing has enabled genetic therapies including an approved treatment for sickle cell disease (1). To advance the utility of genome editing in larger patient populations, efficient methods are needed to deliver editing enzymes into diseased cells in the body. Enveloped delivery vehicles (EDVs) are virally derived particles that can package and transport genome-editing ribonucleoproteins (RNPs) into cells in culture and in vivo (2, 3). These particles are programmable when engineered to display both a fusogen and antibody fragments on their surface (2, 4). While attractive as a delivery strategy for CRISPR-Cas9 RNPs, the structure and delivery mechanism of EDVs have yet to be determined.

Derived from HIV-1 lentiviral vectors, EDVs could employ multiple mechanisms of protein and nucleic acid nuclear delivery. Lentiviral vectors (LVs) package nucleic acids and associated proteins, such as nucleocapsid, into a proteinaceous capsid core structure that assembles during virion maturation (5–8). After virions escape from endosomes in infected cells, the capsid cores protect the RNA genome and associated proteins from innate immune detection, traveling along microtubules to deposit their contents into the nucleus by translocation across the nuclear pore (9–11). Some HIV-1 proteins, including the matrix protein, contain nuclear localization signals (NLSs) that bind to host proteins, such as importin α, for transport through the nuclear pore complex (12). Other HIV-1 proteins, such as the integrase, may use both the capsid and NLSs for nuclear delivery (13). Because the Cas9 RNPs packaged in EDVs comprise both nucleic acids and NLS-containing proteins, their mechanism of EDV-mediated nuclear delivery has been unclear.

Here, we determined the components that are necessary for EDV-mediated genome editing. We found that although the capsid structure assembles in a subset of EDV particles, it does not transport Cas9 RNPs into the nucleus. Instead, NLS peptides engineered into the Cas9 protein confer nuclear entry and can be tuned to improve delivery efficiency. Furthermore, Mechanism-guided engineering enabled simplification of the EDV design, creating miniEDV particles with only 22% of the original viral residues while achieving up to 2.5-fold higher editing potency compared to the original EDVs in primary human T cells. Understanding the functional components of virally derived particles paves the way toward more effective and readily manufacturable genome editing therapies.

## Results

### The EDV Capsid Core Does Not Mediate Nuclear Delivery of Cas9 RNPs.

**Fig. 1.**
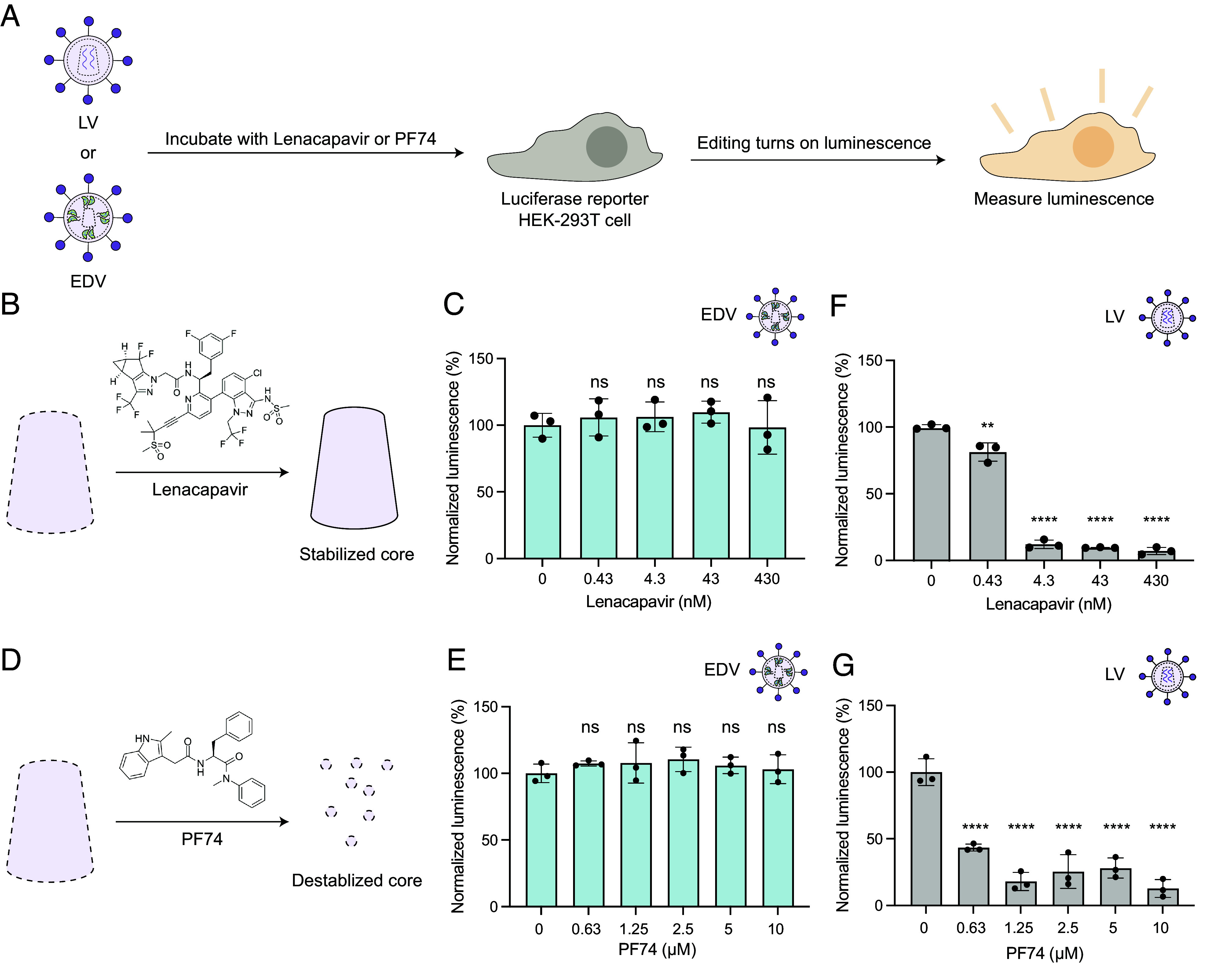
Small-molecule inhibitors that disrupt the capsid core do not impact EDV editing. (*A*) Schematic of small-molecule inhibition experiments. EDVs or LV were incubated with luciferase reporter HEK-293T cells in the presence of lenacapavir or PF74. The luminescence of the reporter cells were recorded after incubation. (*B*) Schematic showing that lenacapavir stabilizes the capsid core. (*C*) Lenacapavir did not inhibit EDVs compared to the DMSO control (0 nM). (*D*) Schematic showing that PF74 destabilizes the capsid core. (*E*) PF74 did not inhibit EDVs compared to the DMSO control (0 nM). (*F*) Lenacapavir inhibited LVs compared to the DMSO vehicle control (0 nM). (*G*) PF74 inhibited LVs compared to the DMSO vehicle control (0 nM). Schematics are not to scale. Data were normalized to the DMSO control. *P*-values were calculated using an ordinary one-way ANOVA with Dunnett’s multiple comparisons test to the vehicle control. Mean ± SD of n = 3 batches of EDVs. Nonsignificance indicated by “ns”, *P ≤ 0.05, **P ≤ 0.01, ***P ≤ 0.001 ****P ≤ 0.0001.

We showed previously that inhibiting the capsid core with a preclinical small molecule, GS-CA1, did not reduce EDV editing activity (2). This preliminary result suggested that the capsid core was not essential for nuclear transport of Cas9 RNPs, a surprising finding given the central role of the capsid structure in LV cargo nuclear localization. In LV, the capsid core packages and facilitates nuclear transport of the nucleic acid and associated protein cargo. To explore this further, we tested two additional small molecule inhibitors of the capsid core, lenacapavir and PF-3450074 (PF74) (Experimental schematic shown in Fig. 1*A*) (14–16). We produced EDVs packaging Cas9 RNPs that cut a prematurely truncated luciferase reporter gene (C205ATC) (17). HIV-1 lentiviral vectors packaging a transgene encoding Cas9 enzymes and the same guide RNA were used as a positive control. The particles were incubated with HEK-293T cells expressing the truncated luciferase reporter in either the presence or absence of the inhibitors. Cutting leads to insertions and deletions that can restore the luciferase reporter reading frame. We found that luciferase expression was specific to cleavage at the luciferase locus, proportional to the dose of EDVs and detectable 48 h after transduction (*SI Appendix,* Fig. S1). In the presence of increasing concentrations of lenacapavir, a clinically approved HIV-1 inhibitor that impairs cargo delivery by stabilizing the core (Fig. 1*B*) (14, 15). no decrease in EDV-mediated induction of reporter cell luminescence occurred (Fig. 1*C*). Similarly, incubation of cells with increasing concentrations of the capsid core destabilizer PF74 (Fig. 1*D*) (16) had no effect on EDV-mediated luminescence (Fig. 1*E*). Parallel experiments with LVs encoding analogous components (Cas9 and sgRNA against the luciferase reporter gene) showed dose-dependent loss of reporter cell luminescence, consistent with inhibitor prevention of nuclear delivery (Fig. 1 *F* and *G*). Together, these results support the conclusion that the capsid core is not needed for Cas9 RNP delivery by EDVs.

As the luminescence produced by the reporter cells depends on both nuclear entry and Cas9 editing, we directly tested whether Cas9 nuclear entry required the capsid core. We incubated HEK-293T cells with EDVs and PF74 for 24 h, isolated cell nuclei and used Western blots to determine the relative amounts of Cas9 enzymes or capsid associated with the nucleus (*SI Appendix,* Fig. S2*A*). PF74 was used in this experiment because lenacapavir has been shown to stall capsid cores on the cytosolic side of nuclear pores, leading to their coisolation with the nuclear fraction (18). We confirmed successful nuclear isolation by monitoring nuclear localization of EZH2 and cytosolic localization of GAPDH (*SI Appendix,* Fig. S2*B*). The 24 kDa mature capsid protein decreased in the nuclear fraction in the presence of 10 µM PF74, while the amount of Cas9 enzyme remained consistent across all PF74 concentrations (*SI Appendix,* Fig. S2*B*). Both the Gag–Cas9 polyprotein (220 kDa) and Cas9 (160 kDa) were present in the nuclear fractions. The presence of Gag–Cas9 in the nuclear fractions is surprising because it was assumed that editing enzymes needed to be liberated from viral structural proteins to enable nuclear entry (2, 3, 19, 20). The observation of Gag associated with the nucleus is consistent with previous reports that the HIV-1 Gag protein can localize to euchromatin and associate with DNA demethylases in the nucleus (21, 22). Our results suggested that the liberation of Cas9 enzymes by protease cleavage may not be necessary for nuclear association. These results confirm that Cas9 RNP delivery into the nucleus is independent of the EDV capsid core.

### EDVs Form Capsid Cores That Do Not Encapsulate Cas9.

**Fig. 2.**
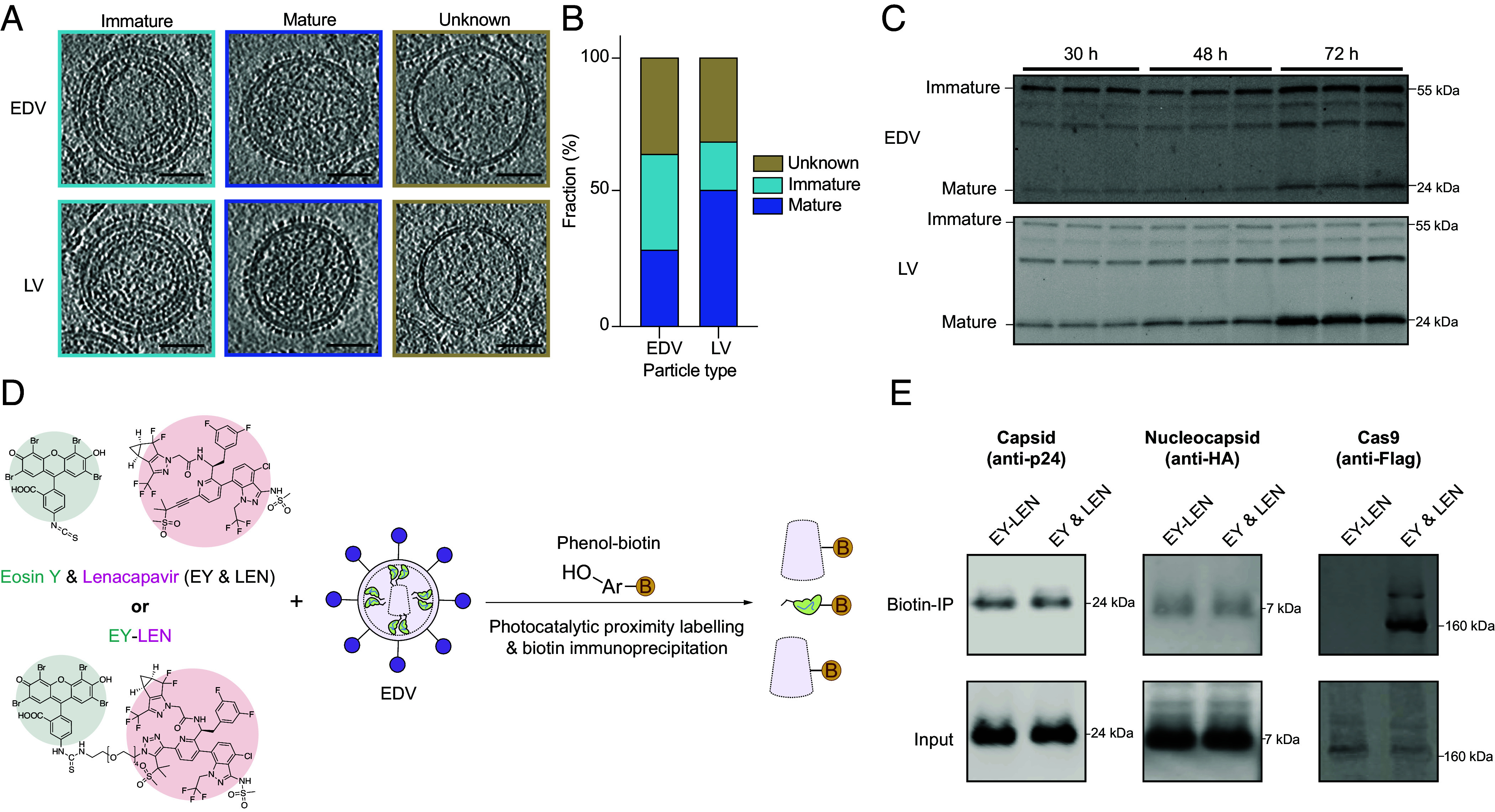
EDVs have capsid cores that do not encapsulate Cas9. (*A*) Representative two-dimensional slices from cryogenic-electron tomograms of EDVs and LVs that are immature, mature, or unknown. (Scale bars are 50 nm.) (*B*) Fraction of EDVs (n = 498) and LVs (n = 374) that are mature, immature, or unknown. (*C*) Western blot showing the fraction of immature capsid protein (55 kDa) compared to mature capsid protein (24 kDa) in EDVs and LVs harvested 30, 48, or 72 h after transfection. Three independent batches of EDVs and LVs were harvested (one per lane). Partially mature forms of the capsid protein are also visible in the blot (bands ~30 kDa). (*D*) Schematic of photocatalytic proximity labeling experiment. The Eosin Y–Lencapavir (EY–LEN) conjugate (500 nM) or unconjugated EY and LEN (EY & LEN, 500 nM each) were incubated with EDVs. Phenol biotin probes were then added to enable biotinylation of proximal proteins upon blue light illumination. Biotinylated proteins were isolated using biotin enrichment (biotin-IP). Schematic is not to scale. (*E*) Western blot showing the amount of biotinylated Cas9, mature nucleocapsid protein, or mature capsid upon photocatalytic proximity labeling. The proximity labeling experiments were similarly repeated twice.

We wondered why Cas9 RNP nuclear entry was independent of the capsid core. We began by testing whether EDVs contained capsid cores as observed in LVs, because the absence of capsid cores could explain the lack of effect from the capsid inhibitors. After purification by iodixanol cushion ultracentrifugation to remove contaminating proteins, EDVs and LVs were visualized using cryogenic electron tomography (23–26). We aligned the tilt movies of the particles, then reconstructed three-dimensional tomograms of our EDV and LVs. Three-dimensional tomograms of the EDVs and LVs (Movies S1 and S2) revealed spherical particles with a lipid bilayer in each case (Fig. 2*A*). Surface glycoproteins appeared as dark spots densely coating the lipid bilayer exterior. We quantified and compared the proportion of mature particles (with a capsid core), immature particles (concentric rings of proteins under the lipid bilayer), and unknown particles. Both EDVs and LVs were of similar size (~125 nm diameter) and contained multiple morphologies of the mature capsid core (*SI Appendix,* Fig. S3 *A–D*) (27). Roughly 29% of EDVs and 51% of LVs contained a capsid core, while 36% of EDVs and 18% of LVs were immature with concentric rings of protein (Fig. 2*B*). The remaining ~30% of particles could not be categorized and were presumed to be other types of vesicles or broken particles (Fig. 2*B*). We confirmed the lower proportion of EDVs containing the mature capsid core compared to LVs by Western blotting the capsid protein inside of the particles. Formation of the capsid core requires the 24 kDa capsid protein to be proteolytically cleaved from the 55 kDa Gag polyprotein. In LVs harvested at 30, 48, or 72 h after transfection, the mature capsid protein was more abundant than the uncleaved Gag polyprotein (Fig. 2*C*). In contrast, a similar analysis of EDVs showed the 55 kDa Gag polyprotein was more abundant than the 24 kDa capsid species at all time points (Fig. 2*C*).

This observation led us to examine whether the differences in the formation of the capsid core were due to structural differences between the immature EDVs and LVs. We used subtomogram averaging and alignment to compare the immature capsid domains of EDVs and LVs to those of published HIV-1 structures (PDB: 5L93). Subtomograms containing the capsid protein were iteratively aligned and then averaged to reconstruct the structure of the immature capsid domains inside the particle. This analysis revealed that the immature capsid domains of both EDVs and LVs matched the HIV-1 structure (PDB: 5L93) (24) with RMS deviations of 1.2 Å and 1.7 Å, respectively (*SI Appendix,* Fig. S3 *E* and *F*). These data show that immature capsid domains in EDVs were structurally indistinguishable from LVs.

We next tested whether EDV editing activity was independent of the capsid core because the core does not encapsulate Cas9. To test this, we used photocatalytic proximity labeling with a eosin Y-lenacapavir conjugate to label proteins located near the capsid core (Fig. 2*D*). We incubated eosinY-lenacapavir or unconjugated lenacapavir and eosin Y with EDVs, then added phenol-biotin (28). Upon illumination with blue light, proteins proximal to the photocatalytic eosin Y were biotinylated and captured by biotin immunoprecipitation. Capsid and nucleocapsid proteins acted as positive controls, because the lenacapavir is bound to the capsid core and the nucleocapsid proteins are located inside of the capsid core. EDVs incubated with unconjugated eosin Y and lenacapavir showed that Gag–Cas9 (220 kDa), Cas9 (160 kDa), nucleocapsid, and capsid proteins could all be biotinylated (Fig. 2*E* EY & LEN lanes), because the eosin Y could diffuse throughout the particle leading to nonspecific biotinylation. When particles were incubated with the eosin Y-lenacapavir conjugate (Fig. 2*E* EY–LEN lanes), both the mature capsid and nucleocapsid protein were biotinylated as expected, because conjugating eosin Y with lenacapavir localizes it to the capsid core. However, we could not detect any Cas9 or Gag–Cas9 proteins. Each sample had similar quantities of input proteins, so the differences in abundance detected by biotin immunoprecipitation were caused by different localization of the photocatalyst and not sample loading. These results are consistent with our observations of uncleaved Gag–Cas9 in the EDVs (2, 3), where the Gag–Cas9 polyproteins are on the inner membrane of the EDVs and distal from the capsid core. This observation shows that small molecule inhibition of the capsid core (Fig. 1) did not decrease the activity of EDVs because Cas9 RNPs do not associate with the core.

### EDV Editing Activity Correlates with NLS Abundance on Cas9.

**Fig. 3.**
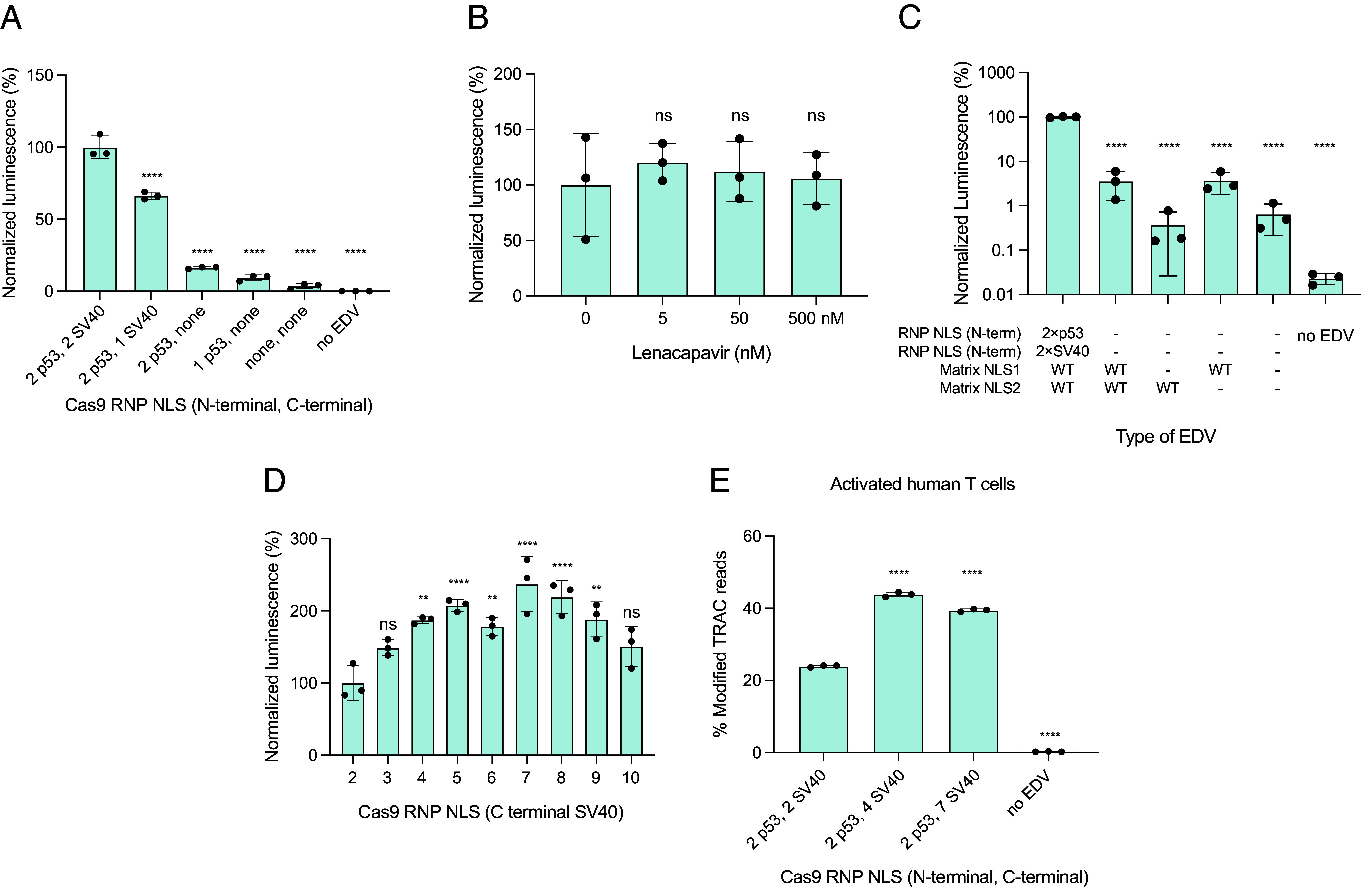
EDV editing activity correlates with NLS abundance on Cas9. (*A*) Removal of NLS on Cas9 enzymes reduced editing and luminescence. Data were normalized to the design with two N-terminal p53 and two C-terminal SV40 NLS. (*B*) The capsid core does not transport Cas9 enzymes missing NLS into the nucleus. EDVs packaging Cas9 without NLS were incubated with luciferase reporter HEK-293T cells in the presence of Lenacapavir (0 to 500 nM). The luminescence of the reporter cells were recorded after 2 d after incubation. (*C*) The NLS in the matrix protein accounts for the residual editing activity. The NLS in the matrix protein were mutated in EDVs packaging Cas9 RNPs without NLS. NLS1 (KKKYK) and NLS2 (KSKKK) were mutated to IIKYK and KSIIK, respectively (29). (*D*) EDV editing correlates with the number of C-terminal SV40 NLS. EDVs packaging Cas9 enzymes with two p53 N-terminal NLS and increasing numbers of SV40 NLS at the C-terminus were created. Data were normalized to the original design, labeled as “2”. (*E*) EDV designs with four or seven C-terminal SV40 NLS increased editing in activated primary human T cells (from one donor) as measured by sequencing at a dose of 4,500 particles per cell. The physical titers of the EDVs were determined using nanoparticle flow cytometry. *P*-values were calculated using an ordinary one-way ANOVA with Dunnett’s multiple comparisons test to the first column in each graph. Mean ± SD of n = 3 batches of EDVs. Nonsignificance indicated by ns, *P ≤ 0.05, **P ≤ 0.01, ***P ≤ 0.001 ****P ≤ 0.0001.

Since the capsid core does not transport Cas9 RNPs to the cell nucleus, we reasoned that engineered NLSs on the Cas9 enzyme might be essential for nuclear entry and editing activity. EDVs were prepared with Cas9 RNP cargo bearing different NLS designs, and equal volumes of these EDVs were incubated with luciferase reporter cells to simultaneously determine differences in particle titer and editing efficiency. We systematically tested different numbers N-terminal p53 NLSs and C-terminal SV40 NLSs on the packaged Cas9 enzymes. NLS reduction corresponded to a decrease in the luminescence of reporter cells, consistent with a requirement for NLS-mediated Cas9 nuclear transport (Fig. 3*A*). Removing the C-terminal SV40 NLS had a larger effect on reporter luminescence than removing the N-terminal p53 NLS, indicating that the type and position of NLS is important for nuclear transport. Removing all NLSs reduced the luminescence of EDV-treated reporter cells by more than 95%. We further tested whether the residual editing activity of the Cas9 RNPs without NLSs could be due to nuclear transport by the capsid core. Lenacapavir did not significantly decrease the luminescence of reporter cells incubated with EDVs packaging Cas9 RNPs lacking NLSs, indicating that the residual editing activity was not due to capsid core transport (Fig. 3*B*). We next tested whether the residual editing activity was due to two naturally occurring NLSs in the matrix protein (29). Mutating the first NLS or both NLSs in the matrix protein in EDVs packaging Cas9 lacking NLSs further decreased the luminescence of the reporter cells from 4 ± 2% to 0.4 ± 0.3%. Mutating the second NLS alone in the matrix protein in EDVs packaging Cas9 lacking NLSs did not have an effect. These results show that the residual editing activity was due to a small amount of Cas9 RNP nuclear transport from the matrix protein.

We also found that adding additional NLSs to Cas9 enhances EDV-mediated Cas9 RNP editing efficiency. We created EDVs packaging Cas9 RNPs containing two to ten SV40 NLSs at the C-terminus of the Cas9 enzyme (Fig. 3*D*), because the C-terminal SV40 NLS had a larger effect on editing (Fig. 3*A*). Cas9s with four to nine NLSs showed ~twofold higher activity compared to the original two-NLS design, with seven NLSs being the best (2). Adding additional N-terminal NLSs to the Cas9 enzymes with seven C-terminal NLSs did not further improve EDV-mediated editing activity (*SI Appendix,* Fig. S4*A*). We note that editing efficiency decreased when excess NLS were added because the expression of the Gag–Cas9 polyprotein in the producer cells decreased, impacting EDV production (*SI Appendix,* Fig. S4*B*). To confirm that the improvements in EDV editing were not specific to the luciferase reporter cells, we tested our best designs (the four- and seven-NLS constructs, Fig. 3*D*) in primary human-activated T cells. We targeted the *TRAC* locus to disrupt the native T cell receptor (TCR), a step in the creation of therapeutic TCR-T cells (30). Activated T cells were incubated with an equal number of EDVs as determined by nanoparticle flow cytometry (31, 32), and editing was quantified 3 d postincubation by sequencing. EDVs packaging Cas9 RNPs with four or seven C-terminal NLSs increased editing by 79% and 73% at the *TRAC* locus, respectively, compared to our initial design (Fig. 3*E*). This increase in *TRAC* editing resulted in a corresponding reduction in the number of TCR-expressing T cells as quantified by flow cytometry (*SI Appendix,* Fig. S4*C*).

### Capsid Core-Related Components Are Unnecessary for EDV Function.

**Fig. 4.**
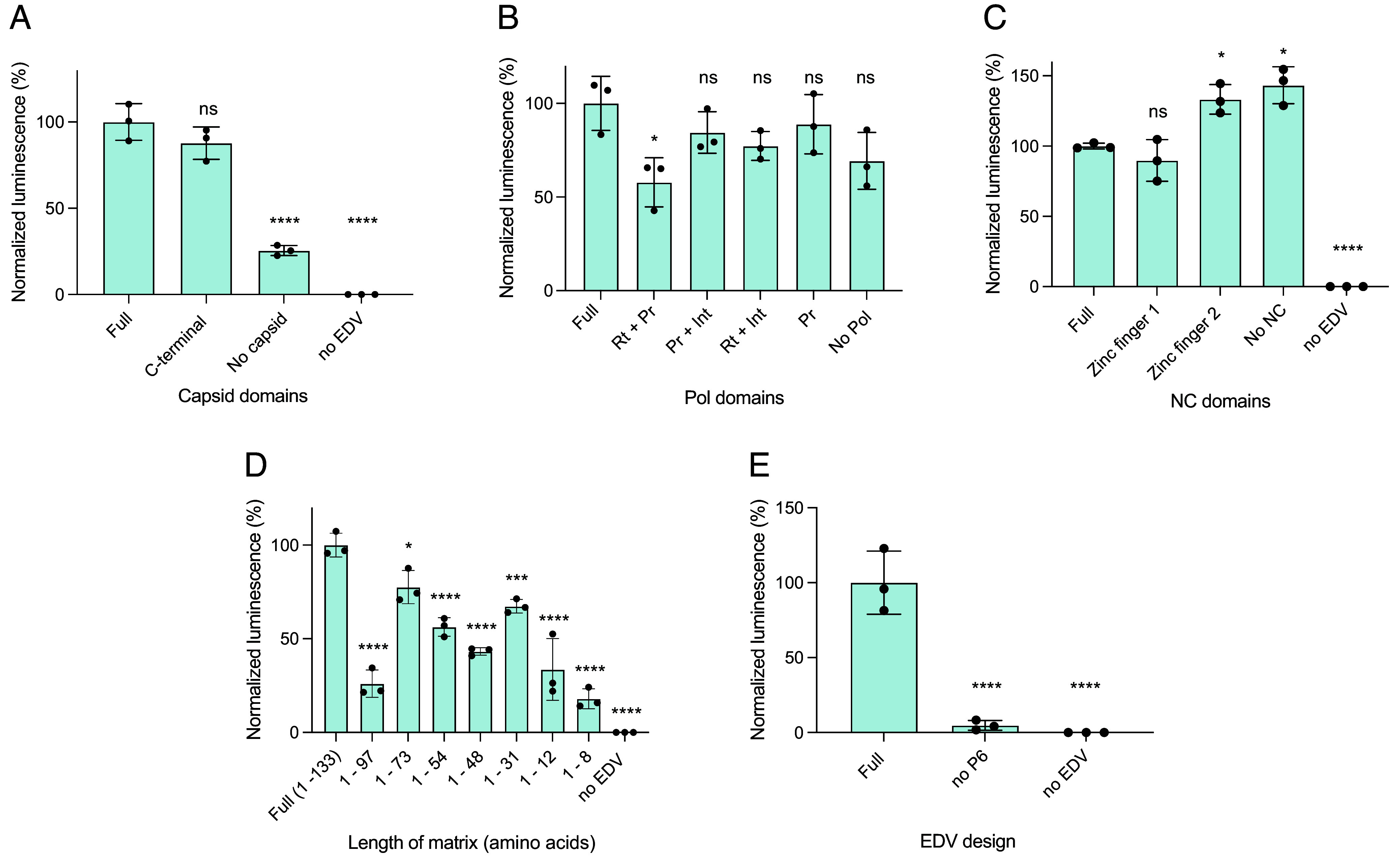
Capsid core-related components are unnecessary for EDV function. (*A*) The C-terminal domain of capsid was sufficient for EDV activity. The N-terminal domain of the capsid protein (residues 5 to 148) or the entire capsid protein (amino acids 5 to 227) was removed. Data were normalized to EDVs containing the full capsid. (*B*) The Pol polyprotein is unnecessary for EDV activity. Domains from the Pol polyprotein were removed systematically and the activity of the EDVs were compared to EDVs containing the full Pol polyprotein. Rt: Reverse transcriptase, Pr: Protease, Int: Integrase. Data were normalized to EDVs containing the full Pol. (*C*) The nucleocapsid (NC) is unnecessary for EDV activity. The zinc fingers in the NC protein were removed and the activity of the EDVs were compared to EDVs containing the full NC. Data were normalized to EDVs containing the full NC. (*D*) The matrix is necessary for EDV activity. The matrix protein was minimized one secondary structure element at a time from the C-terminal end. Data were normalized to EDVs containing the full matrix. (*E*) The p6 is necessary for EDV activity. The p6 protein was removed and the activity of the EDVs was compared to full EDVs. An equal volume of EDVs were incubated per condition. Mean ± SD of n = 3 batches of EDVs. *P-*values were calculated using a one-way ANOVA with Dunnett’s multiple comparisons to the EDV designs containing the full protein (“Full”). Mean ± SD of n = 3 batches of EDVs. Nonsignificance indicated by ns, *P ≤ 0.05, **P ≤ 0.01, ***P ≤ 0.001 ****P ≤ 0.0001.

Having shown that the capsid core was not necessary for EDV function, we next wondered whether viral structural proteins (such as the capsid, Pol and nucleocapsid) that form or interact with the EDV capsid core could be removed. This could simplify particle production and avoid undesirable interactions with host cell proteins, especially as Gag proteins can be found in the nucleus (21, 22, 33, 34). We made deletions to the viral structural proteins in the EDVs, then incubated them with luciferase reporter cells. Equal volumes of EDVs were added to reporter cells to capture both changes in particle production and editing activity. Based on data showing that the C-terminal domain of the capsid protein was sufficient for immature HIV-1 virions assembly (24, 35), we removed the capsid N-terminal domain (amino acids 5 to 148) from the Gag, Gag–Pol, and Gag–Cas9 polypeptides and tested the resulting EDVs in luciferase reporter cells. N-terminal domain removal had no effect but removing the entire capsid protein (amino acids 5 to 227) decreased editing by ~75% (Fig. 4*A*). Next, we tested the removal of the Pol polyprotein, which is composed of the viral protease, reverse transcriptase and integrase. The viral protease matures HIV-1 virions to form the capsid core. It may also liberate Cas9 RNPs from Gag proteins but was previously found to be unnecessary in murine leukemia virus-based particles packaging base editors (36). Reverse transcriptase and integrase assemble with the capsid core to form the preintegration complex with the HIV-1 lentiviral genome for transgene integration (37). As EDVs do not package a lentiviral genome, we hypothesize that the Pol would not be necessary. Removing either the viral protease only or viral protease, integrase and reverse transcriptase did not significantly decrease the activity of the EDVs (Fig. 4*B*). We further tested the removal of the nucleocapsid protein which condenses the viral genome inside of the capsid core (38). and should be unnecessary in EDVs. The nucleocapsid is composed of two zinc fingers. EDVs with only the first zinc finger did not show a reduction in editing. Removing the first zinc finger or removing the entire nucleocapsid protein increased EDV-mediated editing by 43% (Fig. 4*C*).

**Fig. 5.**
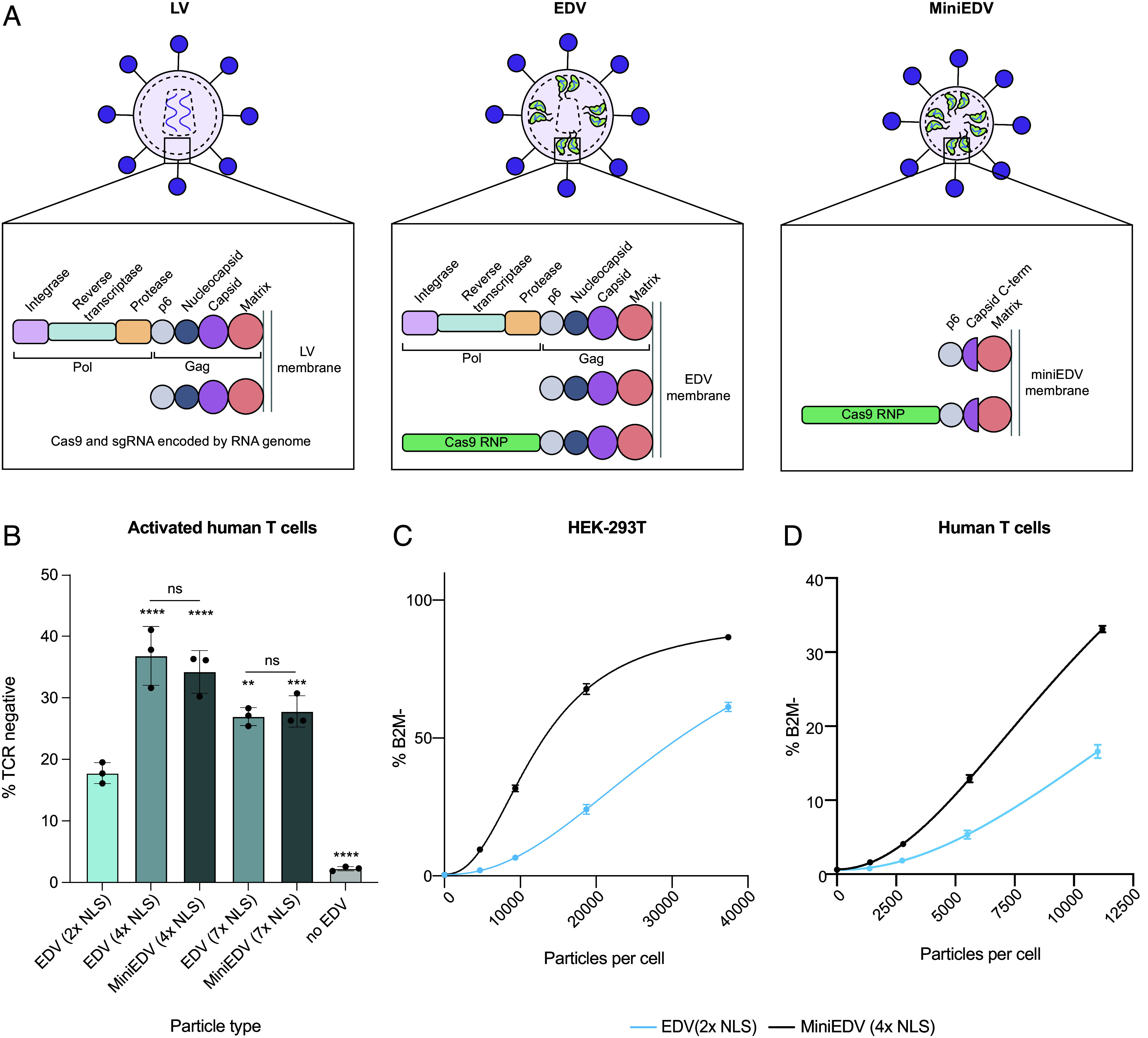
Removing capsid core–related components and optimizing Cas9 nuclear localization created minimal EDVs. (*A*) Schematic showing the viral structural proteins in LVs, EDVs, and minimized EDV (miniEDVs). The schematic is not drawn to scale. (*B*) MiniEDVs had higher activity than the original EDVs (2 × NLS). The indicated particles were incubated with primary activated human T cells (12,000 particles per cell). Particle numbers were determined using the NanoFCM Nanoanalyzer. Expression of the TCR was quantified 5 d after incubation. Mean ± SD of n = 3 batches of EDVs. *P-*values were calculated using a one-way ANOVA with Šídák’s multiple comparison tests between the indicated pairs and the EDV (2 × NLS) design. Nonsignificance is indicated by ns, *P ≤ 0.05, **P ≤ 0.01, ***P ≤ 0.001 ****P ≤ 0.0001. (*C*) Editing at the *B2M* locus in HEK-293T cells was compared between miniEDVs (4 × NLS) and EDVs (2x NLS). Editing was determined 5 d after incubation using flow cytometry. Particle numbers were determined using the NanoFCM Nanoanalyzer. (*D*) Editing at the *B2M* locus in activated T cells was compared between miniEDVs (4 × NLS) and EDVs (2 × NLS). Editing was determined 5 d after incubation using flow cytometry. Particle numbers were determined using the NanoFCM Nanoanalyzer.

We next tested whether the two remaining HIV-1 proteins, matrix and p6, were necessary for EDV function. The matrix protein is myristoylated and anchors the viral structural proteins to the inside of the producer cell membrane to enable particle assembly (38). We also found that the matrix protein contains NLSs that contribute to the nuclear delivery of the Cas9 RNP (Fig. 3*C*). Consequently, we anticipated that it would be essential for EDV function, but previous reports have also suggested that the first eight amino acids of matrix containing the myristoylation signal were sufficient for particle production (39). We tested this hypothesis by truncating the matrix protein one secondary structural element at a time starting from the C-terminus and found that any deletions to the matrix protein decreased EDV activity (Fig. 4*D*). We next tested removing the p6 protein from EDVs. The p6 protein recruits the producer cell’s Endosomal Sorting Complexes Required for Transport (ESCRT) machinery for particle budding (40). Removing the p6 signal ablated the activity of the EDVs (Fig. 5*E*). Altogether, these data show that most viral proteins related to the capsid core (N-terminal of the capsid, nucleocapsid, protease, integrase, and reverse transcriptase) were not necessary in EDVs, but the matrix and p6 proteins were essential.

### Removing Capsid Core-Related Components Created Functional Minimal EDVs.

We combined our core-related deletions and NLS optimizations together to create minimal EDVs (miniEDVs) using only 22% of the viral residues of the original EDVs (Fig. 5*A*). We first used cryogenic electron tomography to confirm that miniEDVs formed particles. Cryogenic electron tomograms showed that miniEDVs were 80 ± 30 nm in diameter, ~25% smaller than the original EDVs (*SI Appendix,* Fig. S5 *A* and *B*). The lipid envelope and glycoproteins were visible. Patches of protein density underneath the membrane were visible that may correspond to the minimized Gag protein (*SI Appendix,* Fig. S5*C*). Having confirmed that miniEDVs formed particles, we next quantified their yield. We tested both four and seven C-terminal NLS Cas9 designs due to their similar editing potency (Fig. 3*E*). We found that producer cells were able to produce an equal number of miniEDVs (~10^9^ particles/mL) compared to EDVs (*SI Appendix,* Fig. S5*D*) using nanoparticle flow cytometry. As the producer cells may also produce other vesicles and exosomes that are morphologically similar to EDVs, we quantified these background particles by transfecting producer cells with noncoding plasmids. Cells transfected with noncoding plasmids produced 2 × 10^8^ particles/mL. These results indicate that ~80% of particles produced were likely to be EDVs and agree with our particle classifications in Fig. 2*B* where ~70% of the particles were EDVs. We further quantified the loading of Cas9 and sgRNA into the particles as this is crucial for editing activity. Using enzyme-linked immunosorbent assays, we found that the EDVs with two-NLS, miniEDVs with four-NLS, and miniEDVs with seven-NLS packaged 470 ± 60, 320 ± 50, and 210 ± 20 Cas9 enzymes respectively (*SI Appendix,* Fig. S5*E*). Quantifying the packaging of sgRNAs using real-time quantitative reverse transcription polymerase chain reactions, we observed that EDVs with two-NLS, miniEDVs with four-NLS, and miniEDVs with seven-NLS packaged 230 ± 30, 199 ± 5, 187 ± 7 sgRNAs per particle respectively (*SI Appendix,* Fig. S5*F*). These results are consistent with previous work showing that the number of sgRNA inside EDVs was limiting for RNP formation (2) and suggest that both EDVs and miniEDVs package roughly 200 Cas9 RNPs per particle. We further found that miniEDVs could be produced without supplementing producer cells with plasmids encoding extra Gag–Pol proteins (*SI Appendix,* Fig. S6), which simplifies their production. In addition, single-chain antibodies can be displayed on miniEDVs to mediate cell entry (*SI Appendix,* Fig. S7). Overall, our results demonstrate that miniEDVs could be created with ~20% of the viral residues without a compromise in particle yield or RNP packaging compared to EDVs.

Finally, we compared the editing efficiency of the miniEDVs to both our original and NLS-optimized EDV designs using activated T cells from second donor. Comparing the two donors, the NLS-optimized EDV designs showed similar increases in editing activity (*SI Appendix,* Fig. S8). EDV-encapsulated Cas9 RNPs targeted the *TRAC* locus, and editing was quantified by measuring the decrease in TCR expression using flow cytometry 5 d postincubation (Fig. 5*B*). MiniEDVs packaging four or seven C-terminal NLS Cas9s increased editing by 107% and 53%, respectively, relative to the original EDVs (2). MiniEDVs had comparable editing to their respective NLS-optimized EDV counterparts, indicating that removing unnecessary viral components did not negatively affect editing efficiency. We further compared the editing efficiency of the miniEDVs packaging four-NLS Cas9s across a range of concentrations in both HEK-293T cells and activated primary human T cells at the *B2M* locus against our previously published best EDV design with 2 × NLS ((2) to benchmark their improvement in editing efficiency. We chose the *B2M* locus, because its disruption enables the production of allogeneic chimeric antigen receptor T cells (41). We observed an average increase in editing per EDV particle of ~2.5-fold in both HEK-293T and activated T cells (Fig. 5 *C* and *D*). Neither the full EDVs or the miniEDVs decreased the viability of the HEK-293T cells or primary human T cells (*SI Appendix,* Fig. S9). This shows that miniEDVs could efficiently edit genomes with minimal cytotoxicity. Ultimately, understanding the EDV components necessary for Cas9 delivery allowed us to simultaneously increase particle editing potency and streamline their production for genome editing.

## Discussion

Virally derived particles, including EDVs, have emerged as promising delivery vehicles for genome editing. EDVs were derived from lentiviral vectors by fusing Cas9 RNPs to the end of the structural Gag protein. Beyond this change, EDVs retained all the same components as second-generation LVs (Fig. 5*A*). Cryo-electron tomography showed that EDVs and LVs share similar morphology and capsid structures. However, unlike LVs, EDVs do not use the internal capsid core for nuclear delivery of Cas9 RNPs. Instead, EDV-mediated genome editing depended on the presence of NLS peptides engineered onto Cas9. Future studies could identify the specific nuclear pore proteins (such as, importin α or nuclear transport factor 2) used by the RNPs to enter the nucleus (42, 43). We also found that Cas9 RNPs are not associated with the capsid core. Removal of capsid-core-related proteins and optimization of Cas9 RNP nuclear localization created simpler and more efficacious miniEDV particles. MiniEDVs showed 2.5-fold higher editing potency relative to our initial EDVs (2) in both cell lines and primary cells. MiniEDVs can be produced in cells transfected with two plasmids, compared to three or more plasmids required for full EDVs.

The miniEDVs are 25% smaller than the full EDVs, yet packaged the same quantity of guide RNAs and by extension Cas9 RNPs. The components of our minimized particles hold important lessons for engineering exosomes and other biological particles. We show that a membrane-binding domain (matrix), an assembly domain (C-terminal CA), and a budding signal (p6) are sufficient for packaging and exporting a proteinaceous therapeutic cargo. We anticipate that miniEDVs could be used beyond genome editing for delivery of enzymes, cytokines, and other therapeutic proteins. MiniEDVs do not contain viral enzymes (protease, reverse transcriptase or integrase) or viral nucleic acid-binding domains (nucleocapsid), reducing the possibility of unwanted interactions with target cells. Future work could focus on understanding the effect of the miniGag proteins on editing specificity and immunogenicity.

While we focused on an HIV-1-derived particle system, we anticipate that other virally derived particles, including those based on related retroviruses, contain unnecessary proteins and could be simplified. As virally derived particles and EDV systems advance toward clinical use, ensuring that these delivery vehicles contain only necessary components is critical to reduce complexity, improve manufacturing pipelines, and potentially reduce immunogenicity. The finding that miniEDVs require fewer plasmids to be produced while exhibiting higher editing activity and programmable cell entry, underscores the value of a mechanism-based approach to development. These results lay the groundwork for creating fully synthetic particles that use viral proteins to facilitate delivery, making genome editing therapies simpler, easier to produce, and more efficacious.

## Materials and Methods

Detailed materials and methods are provided in *SI Appendix*. Briefly, appropriate spacers and modifications were cloned into pJRH-1179 U6-reci Gag–Cas9 v2 (referred to as Gag–Cas9) and pJRH-1180 U6-reci Gag–pol v2 (referred to as Gag–Pol) plasmids using NEBuilder® HiFi DNA assembly. EDVs were produced by transfecting Gag–Cas9, Gag–Pol, and surface protein plasmids in HEK-293 T cells. Particles were harvested 48 h after transfection and cell debris was removed by centrifugation and filtering through a 0.45-μm filter. Particles were additionally purified and concentrated by sucrose or iodixanol cushion ultracentrifugation for cell or structural studies. Particles were also characterized by nanoparticle flow cytometry, Cas9 enzyme-linked immunosorbent assays, and quantitative reverse transcription polymerase chain reactions for the single guide RNAs. EDVs (normalized by particle number or volume) were subsequently incubated with HEK-293 T, luciferase reporter HEK-293 T cells, or activated human T cells in the presence or absence of small molecule inhibitors. Editing was quantified 2 to 7 d after incubation using flow cytometry, luminescence assays, or next-generation sequencing.

## Supplementary Material

Appendix 01 (PDF)

Movie S1. Representative tomogram of EDVs.

Movie S2. Representative tomogram of lentiviral vectors.

## Data Availability

Plasmids generated in this study are available from Addgene (ID: 228957–228960) 44–47. Representative tomograms (EDV: EMD-47705, LV: EMD-47741, miniEDV: EMD-47858) and 3D maps (EDV: EMD-47745, LV: EMD-47743) are available from EMDB 48–52.
